# A Novel Analysis Method for Paired-Sample Microbial Ecology Experiments

**DOI:** 10.1371/journal.pone.0154804

**Published:** 2016-05-06

**Authors:** Scott W. Olesen, Suhani Vora, Stephen M. Techtmann, Julian L. Fortney, Juan R. Bastidas-Oyanedel, Jorge Rodríguez, Terry C. Hazen, Eric J. Alm

**Affiliations:** 1 Department of Biological Engineering, Massachusetts Institute of Technology, Cambridge, MA 02139, United States of America; 2 Department of Civil and Environmental Engineering, University of Tennessee, Knoxville, TN 37996, United States of America; 3 Oak Ridge National Laboratory, Oak Ridge, TN 37831, United States of America; 4 Institute Centre for Water and Environment (iWater), Masdar Institute of Science and Technology, Abu Dhabi, UAE; INRA, FRANCE

## Abstract

Many microbial ecology experiments use sequencing data to measure a community’s response to an experimental treatment. In a common experimental design, two units, one control and one experimental, are sampled before and after the treatment is applied to the experimental unit. The four resulting samples contain information about the dynamics of organisms that respond to the treatment, but there are no analytical methods designed to extract exactly this type of information from this configuration of samples. Here we present an analytical method specifically designed to visualize and generate hypotheses about microbial community dynamics in experiments that have paired samples and few or no replicates. The method is based on the Poisson lognormal distribution, long studied in macroecology, which we found accurately models the abundance distribution of taxa counts from 16S rRNA surveys. To demonstrate the method’s validity and potential, we analyzed an experiment that measured the effect of crude oil on ocean microbial communities in microcosm. Our method identified known oil degraders as well as two clades, *Maricurvus* and Rhodobacteraceae, that responded to amendment with oil but do not include known oil degraders. Our approach is sensitive to organisms that increased in abundance only in the experimental unit but less sensitive to organisms that increased in both control and experimental units, thus mitigating the role of “bottle effects”.

## Introduction

### Paired-sample microbial ecology experiments

Many microbial ecology experiments use amplicon-based sequencing (e.g., 16S rRNA gene sequencing) to study the dynamics of a microbial community, whose members are typically grouped into operational taxonomic units (OTUs). A straightforward experimental design uses control units (i.e., subjects, microcosms, or animals not subjected to the treatment) and pretests (i.e., measurements of microbial community composition taken before the treatment is applied to the experimental units). This two-timepoint, paired-sample design is intended to identify changes in community composition that are specific to the experimental treatment, rather than all the changes that occur in the experimental unit, some of which might be irrelevant to the intentionally applied treatment. For example, aquatic microbial communities transplanted into microcosms often suffer dramatic changes in community composition on account of the transplantation. These extraneous community composition changes are called “bottle effects”. Over the course of the experiment, bottle effects are entangled with the effects caused by the experimental treatment [1]. A similar complication can occur in experiments that study animal-associated microbiota, where changes in community composition can be caused by, for example, interactions with experimenters or circulation of a microbe in the animal facility.

If an experiment is performed with many replicates, there are statistical methods that can make statements about whether the observed changes between the groups were meaningful with respect to a null hypothesis. In some cases, an experiment is very preliminary or the samples are so precious that robust replication is not feasible. For example, we intended the test the effect of crude oil on an ocean microbial community and did not have enough of the sample material, which was collected on a special cruise, for many replicates. Without sufficient replications, it is impossible for us to make a statement about the statistical significance of the results of such an experiment, but we wanted to obtain as much information as possible from such difficult-to-acquire samples, with the caveat that any conclusion would need a separate, properly-powered experiment for verification.

Because of the complications in paired-sample experiments in general and our experimental setup in particular, we aimed to create a method to visualize and generate hypotheses about the dynamics of microbial communities in paired-sample experiments with few or no replicates. Most popular tools for analyzing the results of microbial ecology experiments have shortcomings or complications when applied to this particular experimental setup. We review some classes of these existing tools below.

For convenience, we refer to the four samples in a two-timepoint, two-unit experiment as “control-before” (control unit, pretest sample), “control-after” (control unit, post-treatment sample), “experimental-before”, and “experimental-after”.

### Existing analytical techniques

#### Ordination techniques

Ordination techniques include principal component analysis (PCA), multidimensional scaling (MDS), redundancy analysis (RDA), and canonical analysis of principal components (CAP) [2, 3]. Although very useful for analyzing and visualizing the relationships between many samples, ordination techniques are not easy to interpret in the context of a paired-sample microbial ecology experiment. In an experiment with no replicates, an ordination plot will only have four points, making it difficult to visually or analytically extract interesting information about the community’s dynamics.

#### Clustering techniques

In the context of microbial ecology, clustering techniques like hierarchical clustering or *k*-means clustering use a dissimilarity metric to infer which samples in a data set group together [2]. Clustering techniques are often combined with ordination techniques and give similar insight. They therefore suffer similar drawbacks when analyzing the results of paired-sample experiments. In the case of a single paired-sample experiment with no replicates, there are only four samples, so a clustering technique can only produce a limited number of analytical outcomes.

#### Beta diversity and other tests

Statistical tests like ANOSIM [4] and PERMANOVA [5] are designed to evaluate whether some set of samples in a data set are more similar to one another than they are to other samples in the data set. In general, rigorous statistical inference and machine learning methods require many replicates, which might be impractical for exploratory studies. For example, La Rosa *et al*. [6] calculate that at least 25 samples are needed to determine if two groups of human microbiome samples have meaningfully different compositions at 90% power.

#### Indicator species techniques

Indicator species techniques like IndVal [7] are intended to identify species that especially informative with respect to the ecological community’s composition or abiotic context [8]. Indicator species techniques treat each sample as an independent community rather than, as we consider, timepoints from the same community. For example, if an OTU is rare in the control-before and control-after but abundant in the experimental-before and experimental-after, an indicator species approach would identify that OTU as important to distinguishing the experimental and control *units*, which, although true, does not correctly reflect that that OTU is probably not affected by the experimental treatment, since it changed in neither the control or experimental unit.

#### OTU-by-OTU techniques

There are techniques that can identify OTUs whose dynamics merit further investigation, even if those OTUs are not abundant enough to appear in a community composition chart or if there are not enough replicates for statistical inference. For example, OTUs can be ordered according to their change in relative abundance between the pretest and the post-treatment sample. However, unintuitive signals, called “compositional effects”, can arise when standard analytical techniques are used with compositional data sets like OTU count data [9, 10]. In an aquatic microcosm experiment, for example, a single organism might bloom in response to the experimental treatment, causing an organism with constant absolute abundance to decrease in relative abundance. The reverse is also possible: an organism with constant absolute abundance increases in relative abundance when other organisms decrease in absolute abundance.

### Opportunity for a distribution-based technique

To incorporate controls and pretests, it is important to be able to meaningfully compare OTUs’ abundances across samples. As discussed above, relative abundances are susceptible to compositional effects. In contrast, nonparametric analysis, which uses only the ranks of abundance-ordered OTUs, is robust to compositional effects but loses much of the quantitative information encoded in the relative abundances. For example, an arbitrarily large change in the abundance of one OTU can cause arbitrarily large changes in the relative abundances of all OTUs but will only change the ranks of each OTU by, at most, one. Using ranks presents a tradeoff between robustness (i.e., each rank changing by at most one) and loss of quantitative information (e.g., if the most abundant OTU doubles in abundance, no ranks change). Ranks are also challenging to use with OTU count data because many OTUs have the same number of counts.

If OTU abundances were distributed in a way that could be reliably modeled, a compromise between relative abundances and ranks would be possible. For example, if one organism blooms and all others remain at constant absolute abundance, the overall shape of the distribution of abundances would change very little. As in an analysis that uses relative abundances, the blooming OTU would be registered because that OTU would move up in the distribution. As in an analysis that uses ranks, the unchanging OTUs would remain at the same places in the distribution even though their relative abundances decreased.

### Our method

In this paper, we present an analytical method designed to measure the dynamics of OTUs across two timepoints, to correct for bottle effects using control units, and to correct for unit-specific effects using pretests. The method is framed in terms of an abundance distribution from macroecology research, the Poisson lognormal distribution, that we found accurately models the abundance distribution of OTU count data. As a test of the method’s validity, we show that, in the context of a bioreactor experiment, this method reports that OTUs in microbial communities derived from the same inoculum and subjected to strong but identical conditions have well-correlated responses.

We used our method to identify OTUs in a complex ocean water community, collected off the Egyptian coast, that respond to amendment with crude oil. Most of the sequences we identified classified as *Maricurvus*, *Pseudomonas*, *Alcanivorax*, *Methylophaga*, and Rhodobacteraceae. These clades include known oil degraders as well as organisms that other microbial ecology experiments have suggested may degrade oil. These results demonstrate that our method will be useful for visualizing the effect of the treatment of interest on all OTUs and for quantifying dynamic changes in abundance in a paired-sample microbial ecology with few or no replicates, although properly-powered follow-up experiments would be needed to verify any of these dynamics.

## Materials & Methods

### Poisson lognormal distribution

#### Theory

Noting that many microbial communities are structured by complex ecological processes, we searched for an ecologically-motivated probability distribution that accurately models the abundance distribution of OTUs in natural microbial communities. The truncated Poisson lognormal (TPL) distribution is an attractive candidate. When a value is drawn from the TPL distribution, a true abundance *λ* is first drawn from a lognormal distribution (with scale parameter *μ* and shape parameter *σ*). Then a random integer is drawn from a Poisson distribution with mean *λ*. If the integer is zero, a new *λ* is drawn and is used to draw a new integer.

Among the many models of species-abundance relationships (e.g., [11, 12] and references therein), there is evidence and theory suggesting that fractions of the total niche space allotted to each organisms are approximately lognormal-distributed [13], and the Poisson distribution is a straightforward model for converting a continuous value *λ* into a random number of discrete counts. The Poisson lognormal has been used to model abundance distributions for plants and animals [14–16] and has been used in at least one study [17] that simulated microbial abundances. In most of these applications, the distribution is truncated at zero counts, since, in most cases, it is impossible to distinguish if a species is absent or if it present but very rare; in both cases, that species would present zero counts.

In a microbial ecology context, the TPL framework asserts that the abundances of microbial species in an environment are lognormal-distributed, that is, that the logarithms of those abundances have a Gaussian distribution. The framework also asserts that sequencing produces an integer number of reads for each species. The number of sequencing counts for a species with true abundance *λ* is drawn from a Poisson distribution with mean *λ*. The parameter *μ* is related to the mean of the abundances of microbial species in that environment (conditioned on the depth of sequencing). The parameter *σ* describes the variability of those abundances.

#### Metrics

Once fit to the OTU abundances in a sample, the TPL distribution provides transformations from raw OTU counts to two different values. As mentioned above, in the TPL framework, OTUs’ true abundances *λ* are assumed to be lognormal-distributed with scale *μ* and shape *σ*. If the Poisson lognormal distribution is an accurate model for OTU abundance distributions, samples might have differing parameters *μ* and *σ*, but the underlying distribution of (log *λ* − *μ*)/*σ* should be similar across samples. We expect this because if, roughly speaking, *λ* is lognormal distributed, then log *λ* is normally distributed, and (log *λ* − *μ*)/*σ* accounts for the differences in means and shapes of the normal distribution. An OTU’s number of reads *r* is the maximum likelihood estimator of its true abundance *λ*, so we define the *normalized reads z* ≡ (log *r* − *μ*)/*σ*, which estimates (log *λ* − *μ*)/*σ*.

Given this metric *z* that can be compared across samples, we looked for a sensible way to combine these values as a measure of dynamics. To quantify an OTU’s dynamics between the two timepoints, we define Δ*z*, the change in rescaled reads. This metric is similar to the log fold change in relative abundance, an application of the more general log-ratio transformation commonly used with compositional data sets. To show this connection, consider two samples *i* ∈ {0 = before, 1 = after} from one microcosm, either control or experimental, with the TPL fit parameters *μi* and *σi*. One OTU of interest has counts *ri* in the two samples. In this case,

$$\Delta z\equiv \frac{\text{log}r_{1}-\mu_{1}}{\sigma_{1}}-\frac{\text{log}r_{0}-\mu_{0}}{\sigma_{0}}=\frac{\text{log}r_{1}}{\sigma_{1}}-\frac{\text{log}r_{0}}{\sigma_{0}}+\text{constant}.$$

For comparison, the log fold change in relative abundance is

$$\text{log}\left(\frac{r_{1}/N_{1}}{r_{0}/N_{0}}\right)\;=\text{ log}r_{1}-\text{log}r_{0}+\text{constant,}$$

where *Ni* is the total number of reads in sample *i*. If *σ*_0_ ≈ *σ*_1_, then

$$\Delta z\approx \frac{\text{log fold change}}{\sigma_{0}}+\text{constant,}$$

that is, Δ*z* and the log fold change are approximately linearly related.

Aside from rescaled reads, the TPL distribution can also be used to transform raw OTU counts to the value of the cumulative distribution function *F*, which has the common range [0, 1] across all samples. Having fit the parameters *μ* and *σ*, an OTU with *r* reads has $F(r)\equiv \sum_{r^{'}\leq r}f_{\text{TPL}}(r^{'})$, where *f*_TPL_ is the probability distribution function for the TPL distribution. Conveniently, Δ*F* is defined for all *r*_0_ and *r*_1_, while Δ*z* and the log fold change are poorly defined when *r*_0_ = 0 or *r*_1_ = 0.

#### Implementation

We implemented the TPL fitting, computation of *z* and *F*, and basic visualizations in an R package *texmexseq*, which we have posted at CRAN (cran.r-project.org). Our package is based on the poilog package [18], to which we add convenience functions for interacting with OTU tables, fitting the distribution to multiple samples, and visualizing Δ*z* and Δ*F*.

### Bioreactor experiment

#### Experimental design

We performed a bioreactor experiment to verify that our analytical method would identify similar dynamics in identically-treated microcosms. Briefly, three identical anaerobic serum bottles were loaded with sterile anaerobic media, glucose, and anaerobic sludge from Al Mafraq wastewater treatment plant (Abu Dhabi Sewage Services Company, Al Dhafrah, Abu Dhabi, UAE). Glucose was the only carbon source. Each bioreactor was incubated at 35°C for 48 hours, at which point the bioreactor’s contents was spun down and the cell pellet resuspended in fresh media. This process was repeated 7 times. The timepoints analyzed in this study are the initial inoculum’s community and the bioreactor’s final community (i.e., timepoint 7).

#### Experimental protocol

Inocula were stored at 4°C before starting experiments. Fermentations were carried out in 150 mL serum bottles with a working volume of 60 mL. Anaerobic serum bottles were loaded with media, described below, and sludge. The initial biomass concentration for all the three inocula was 10 g/L dry weight matter. Sterile media consisted of 5 g/L glucose (autoclaved separately from mineral media) and phosphate buffer (0.2 g/L Na_2_HPO_4_·2H_2_O and 2.5 g/L KH_2_PO_4_) diluted on basal anaerobic media [19]. Media pH was adjusted to 5.5 with 1 M HCl.

After inoculation, each bottle was crimped using sterile rubber stoppers and flushed with pure N_2_ for 2 minutes using sterile 0.45 μm pore gas filters. Bottles were incubated immediately after flushing. To re-suspend the inoculum, the broth was centrifuged in sterilized containers at 5,000 g for 5 minutes. The resulting pellets were re-suspended in 60 mL of fresh media. Bottles were again crimped and flushed. Inoculation and media replacement were all performed in a UV-sterilized laminar flow chamber.

DNA from the bioreactors was extracted with MO BIO Ultra Clean Soil DNA isolation kit according to the manufacturer’s protocol. Paired-end Illumina sequencing libraries were constructed using a two-step 16S rRNA PCR amplicon approach described in Preheim *et al*. [20]. Libraries were multiplexed and sequenced on an Illumina MiSeq with paired-end 150 bp reads.

#### 16S data processing

Only forward reads were used in the analysis. Primers were trimmed from the reads by searching for the best-matching position in the read’s first 20 bases, allowing a maximum of 2 mismatches between the primer sequence and the read sequence. Reads that did not match the primer were discarded. Reads were demultiplexed by assigning reads to the best-matching barcode sequence, allowing no more than 1 mismatched base. Reads with no acceptable barcode match were discarded. Reads were trimmed to 120 bases. Shorter reads were discarded. Reads with an expected number of errors, as calculated by Edgar & Flyvbjerg [21], greater than 1.0 were discarded. The sequence data for these experiments are in S1 and S2 Datasets.

### Aquatic microcosm experiment

#### Inoculum collection

Water samples were collected on 12/13 October 2012 aboard MV *Fugro Navigator* in the West Nile Delta region of the Nile Deep Sea Fan from a station (29.571° E, 31.813° N) with a sea floor depth of 1230 m. This work was conducted in BP’s West Nile Delta Concession as part of a larger survey of Eastern Mediterranean ocean microbiology described elsewhere [22]. No specific permits were required for collection of these samples. These field studies did not include the collection of any endangered or protected species. Temperature, salinity, depth, and dissolved oxygen were measured through the water column with a Valeport Midas+ CTD. Samples were collected with Niskin bottles from four depths selected in consideration of differences in temperature, salinity, and depth: within the thermocline (50 m), within an area of increased salinity in the water column (250 m), two-thirds of sea floor depth (824 m), and 20 m above the sea floor (1210 m). Water was removed from the Niskin bottles and stored in pre-cleaned amber glass bottles at 4°C until the microcosms were set up. In this paper, we analyze the result of the experiment performed using water from 824 meters depth.

#### Microcosm design and sampling

We performed a microcosm experiment to evaluate the effect of crude oil on the microbial community in those water samples. 2 L of water from each depth was used for microcosms, 1 L each for a control microcosm and experimental microcosm. Microcosms were incubated at room temperature in amber glass bottles wrapped in tin foil. The experimental microcosms were treated with 100 ppm v/v crude oil. The oil, Norne Blend, was selected because we expected it would be similar in composition to oil in natural reservoirs near the sampling site. When we sampled, there were no wells near the sampling site that were producing oil.

At 0 and 72 hours after the microcosms were prepared, 100 mL subsamples were extracted and immediately filtered through a 0.2 μm filter. DNA was extracted from the filters according to the standard protocol for the MoBio PowerWater DNA Isolation Kit. We amplified a subunit of the V4 region of the 16S rRNA gene following the procedure described in Preheim *et al*. [20]. Extracted DNA was amplified using custom barcoded primers and sequenced with paired-end 250 bp reads on an Illumina MiSeq.

#### 16S data processing

Primers were trimmed from the forward and reverse reads by searching for the best-matching position in the read’s first 20 bases, allowing a maximum of 2 mismatches between the primer sequence and the read sequence. Read pairs without matching forward and reverse primers were discarded. Reads were demulitplexed by assigning reads to the best-matching barcode sequence, allowing no more than 1 mismatched base. Reads with no acceptable barcode match were discarded. Reads were merged by (i) evaluating alignments that would produce merged reads of 263 ± 5 bases, (ii) selecting the alignment with the greatest number of matching bases, (iii) assigning consensus bases and quality scores according to Edgar & Flyvbjerg [21]. Merged reads with more than 2.0 expected errors were discarded. Taxonomic information was collected using the RDP classifier [23] and, in select cases, NCBI BLAST [24]. We searched for chimeras in the data by performing UCHIME with the Broad gold database [25], UCHIME with the RDP training database (version 9), and *de novo* UPARSE at 99% identity [26]. The sequence data for these experiments are in S3 and S4 Datasets and at MG-RAST [27] under accession 4685010.3.

#### OTU calling

For most analysis of the ocean and sludge experiments, we use unique sequences as OTUs (i.e., these are 100% identity OTUs). For visual clarity, we used 99% *de novo* clustering with UPARSE for all samples in Fig 2. In S1 Fig, we called OTUs using multiple methods. To call OTUs with RDP, we truncated every sequence’s taxonomy at the first level that has less than 80% bootstrap confidence, and two sequences that have the same truncated taxonomy are placed in the same OTU. To call reference-based OTUs, we used the Greengenes reference database [28] (August 2013 97% OTUs) and global usearch [29] with minimum 97% identity.

#### Phylogenetic tree

The sequences shown on the tree are the 303 sequences most abundant in the four microcosm experiments. Sequences were aligned to the Greengenes core set [28] using Pynast [30]. One sequence (#229) that did not align to the core set was excluded. The tree was constructed with FastTree [31] and drawn with APE [32].

### HMP samples

The Human Microbiome Project [33] samples were downloaded from the 16S rRNA trimmed data set (HM16STR). Reads from the stool sample (#700014956) and vaginal sample (#700016101) were trimmed to 200 bp. Only V3-V5 region reads were used.

## Results

### Disparate dynamics in control and experimental microcosms

**Fig 1:**
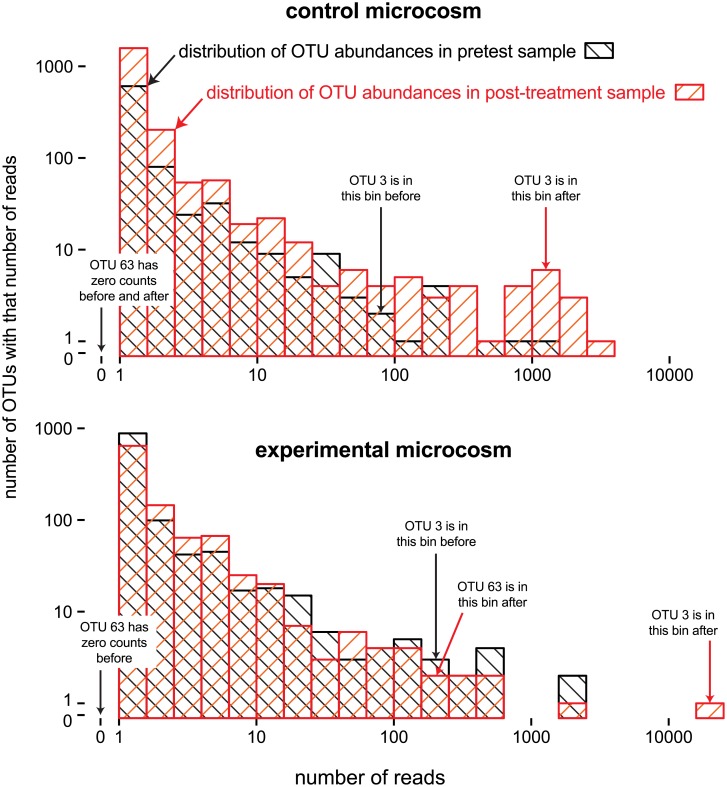
OTU dynamics and possible bottle effects in a paired-sample experiment. Four histograms are shown, one each for each sample in the experiment: control-before (above, black), control-after (above, red), experimental-before (below, black), experimental-after (below, red). Each histogram shows how many OTUs (logarithmic y-axes) have what number of associated reads (logarithmic x-axis). No bin is shown for OTUs with zero counts. The dynamics of two OTUs are shown: black arrows point to the abundance bin for OTUs in the “before” sample and red arrows point their abundance bins in the “after” samples.

Fig 1 shows data from our paired-sample aquatic microcosm experiment. These data highlight some of the issues mentioned in the Introduction. One of the OTUs (OTU 3) increases to a high abundance in the experiment-after sample, potentially introducing a large compositional effect. Furthermore, that OTU’s apparently dramatic dynamics in the experimental microcosm should be treated with skepticism because OTU 3 also increases in the control unit. In contrast, another OTU (OTU 63) was not detected in the two control samples or in the experiment-before sample, but it has a high abundance in the experiment-after sample, suggesting that little or none of its increase in the experimental microcosm should be attributed to bottle effects. How should we compare an OTU’s abundances in the four samples? How should we correct results of the experimental unit with information from the control unit? We were motivated to develop a method based on the Poisson lognormal distribution to answer these questions.

### Poisson lognormal distribution accurately models 16S abundances

**Fig 2:**
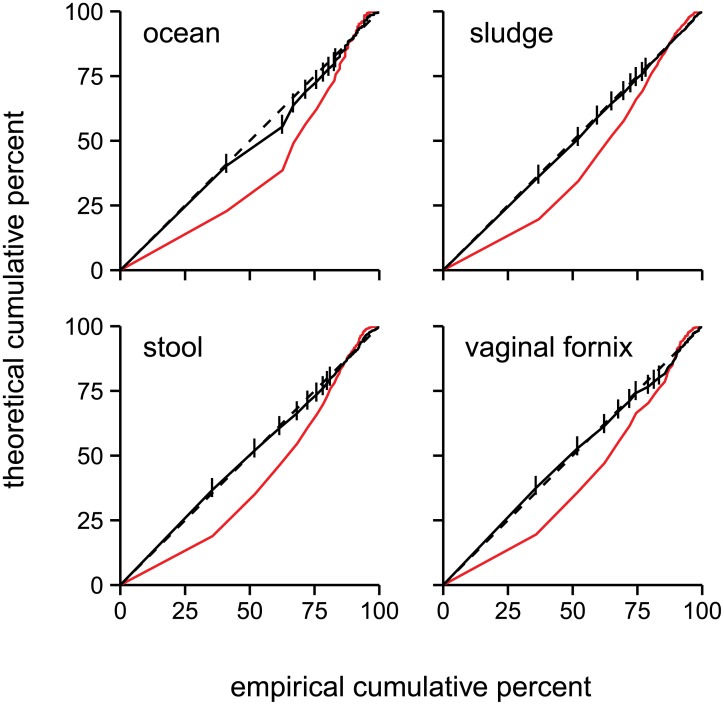
TPL distribution fits OTU abundance distributions in multiple ecosystems. Probability-probability plots comparing the empirical cumulative distribution function (horizontal axis) with the theoretical cumulative probability of a TPL distribution fit to each data set (vertical axis, black solid line). The first ten data points are marked with vertical dashes: the first dash (furthest lower left) represents the fraction of OTUs that have 1 read, the second dash represents the fraction of OTUs with 2 or fewer reads, and so forth. The dotted black line indicates a perfect fit of the TPL to the empirical distribution (*y = x*). The theoretical cumulative probability of a simple lognormal distribution (red line) is shown to emphasize the quality of the TPL fit. The ecosystems are ocean water from this study (top left), wastewater sludge from this study (top right), human stool (bottom left; Human Microbiome Project [HMP] sample), and human vagina (bottom right; HMP sample). 99% *de novo* OTUs are shown for all samples.

We found that the truncated Poisson lognormal (TPL) distribution is an excellent fit for the abundance distributions of OTUs from multiple environments (Fig 2). To quantify the quality of the fit, we conducted an empirical test to see if the differences between the theoretical and observed abundances can be attributed to chance. For each sample shown in Fig 2, we fit the Poisson lognormal distribution to the sample’s data, simulated 10 000 datasets (each with a number of OTUs equal to the number in the observed data) using the fit parameters, and compared the chi-square goodness-of-fit statistic in these simulations to the goodness-of-fit in the observed data. In all cases, the differences between the theoretical and observed distributions are attributable to chance (*p* = 0.76, 0.91, 0.66 for the first three panels in Fig 2), although the attribution to chance was marginal in the sample obtained from a microcosm after treatment with oil (*p* = 0.054; “oiled ocean” in Fig 2). The TPL distribution’s fit is similar when the OTUs were called with some other common OTU-calling methods (S1 Fig).

### Replicate units yield well-correlated results

**Fig 3:**
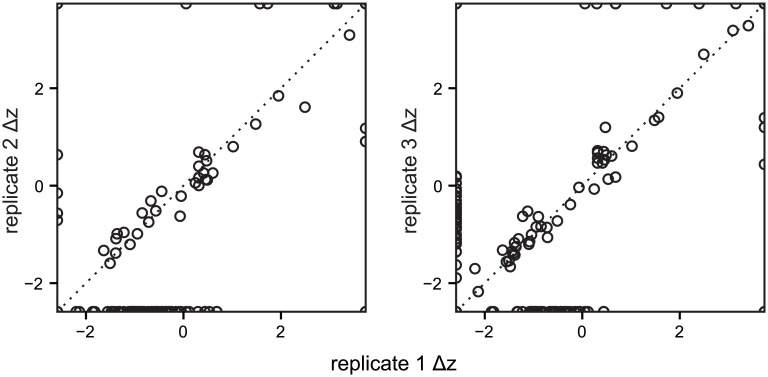
OTUs in replicate units have correlated dynamics. The dynamics of OTUs (circles) in three replicate bioreactors (replicate 1, x-axis; replicates 2 and 3, y-axes) inoculated with the same material and subjected to the same conditions. The dotted line (*y = x*) indicates a perfect correlation: an OTU on this line would have exactly the same Δ*z* in both replicates, while deviations show differences in dynamics. For example, in the left plot, OTUs above the dotted line experienced a greater increase in abundance in replicate 2 than in replicate 1 (or, a smaller decrease in 2 than in 1), while OTUs below the line “grew more” in replicate 2 than in replicate 1 (or, “died less” in 2 than in 1). OTUs with infinite Δ*z* are plotted on the plot’s borders (e.g., the points in the lower- right corner of the first plot represent OTUs that have Δ*z* = +∞ in replicate 1 and Δ*z* = −∞ in replicate 2).

To check that the TPL distribution can be used to quantify dynamics, we compared replicates from the bioreactor experiment, which subjects a microbial community to strong selective pressures. We expected that strong selective pressures would cause dramatic changes in microbial community composition but that these effects would be similar across replicates. Analytically, this means that we expect that Δ*z*, a measure of an OTU’s dynamics, should be similar across replicates. Visually, this means that, if the Δ*z* values for all OTUs in two replicates are plotted against one another, they should fall along the *y = x* diagonal. In fact, the replicated bioreactors show these sorts of well-correlated dynamics (Fig 3). Each plot provides an immediate summary of the relationship between the dynamics of all OTUs in the four samples in the experiment.

### Identification of known and putative oil-degrading organisms

Having shown that metrics derived from the TPL distribution can be used to quantify dynamics, we analyzed the results of an aquatic microcosm experiment with one control microcosm, one experimental microcosm, and two timepoints (pretest and post-treatment samples). In this experiment, ocean water was treated with crude oil to gain insight into the effects of a potential oil spill in this region. Previous work has shown that, in many aquatic environments, a few species (especially in the genera *Alcanivorax* and *Cycloclasticus*) multiply to make up the majority of microbes after crude oil is added [34, 35]. We aimed to identify OTUs in this ecosystem that respond to amendment with crude oil.

To identify OTUs with suggestive responses to oil, we selected criteria for Δ*z* and Δ*F* that would be consistent with a response to oil and not growth due to bottle effects. Specifically, we considered OTUs whose Δ*z* in the experimental unit was greater than or equal to its Δ*z* in the control unit minus 0.5. Roughly speaking, this selected OTUs whose abundances increased more in the experimental unit relative to the control unit. We selected this cutoff criterion because it was compatible with our expectations about specific responses to oil and also included some OTUs with finite Δ*z* values (S2 Fig). Analogous to the fold-change cutoffs used to identify interesting spots on microarrays, this sort of cutoff criterion does not necessarily OTUs whose dynamics would be considered statistically significant in a well-powered experiment.

**Fig 4:**
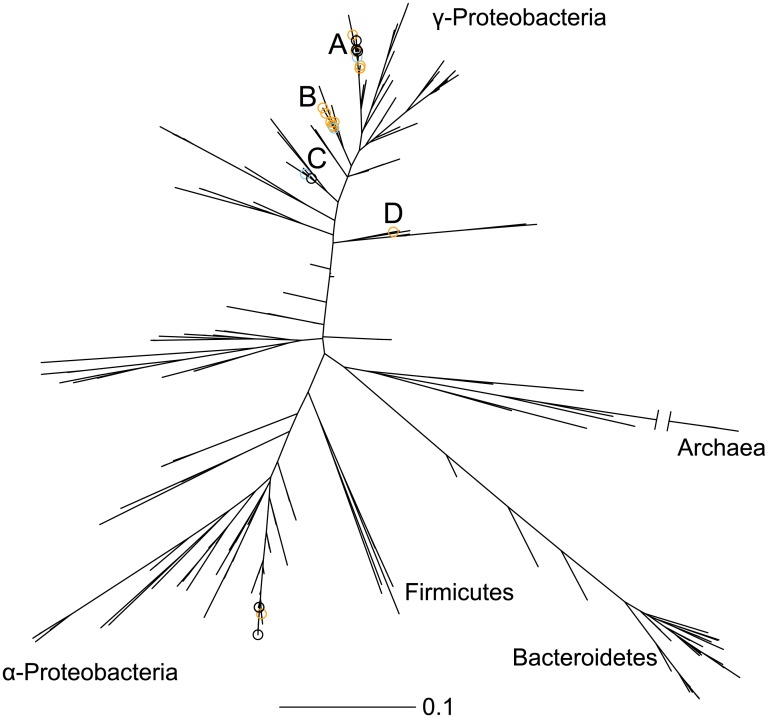
OTUs that respond to oil appear in five clades. On a phylogenetic tree built from the 16S sequences, organisms potentially responding to crude oil are marked with open circles. OTUs that satisfy the Δ*z* criteria are marked with blue circles, OTUs that satisfy the Δ*F* criteria are marked with orange circles, and OTUs that satisfy both are marked with black circles. Information about the taxonomy and dynamics of these sequences are shown in Table 1. The five clades (A through E) are labeled, and select taxonomic groups are labeled to help orient the reader. The Archaea branch is truncated. Scale bar: substitutions per site.

We also considered OTUs whose Δ*F* in the treatment unit was greater than 0.5 but whose Δ*F* in the control unit was less than 0.5. Roughly speaking, this selected for OTUs whose position in the TPL distribution moved *up* past about 50% of other OTUs in the experimental unit but whose position in the distribution moved *down* past about 50% of OTUs in the control unit. A plot of the Δ*F* values which highlights the OTUs that meet the criteria are shown in S3 Fig. The OTUs that satisfy either the Δ*z* or Δ*F* criteria are shown in the context of a bacterial phylogeny in Fig 4. They group into five monophyletic clades. Detailed information about the OTU’s taxonomic classification and dynamics are reported in Table 1.

**Table 1:** OTUs with dynamic behavior in response to amendment with oil.

			criteria		Δr.a.		Δ*z*		Δ*F*		
Clade	Classification	support	Δ*z*	Δ*F*	ct	ex	ct	ex	ct	ex	OTU ID
**A**	*Maricurvus*	0.95	*		0.0330	0.7390	1.382	1.419	-0.0002	0.0041	3
	*Maricurvus*	0.87		*	0.0000	0.0080	n.d.	∞	0.0000	0.9958	63
	γ-Proteobacteria	1.00		*	0.0000	0.0033	n.d.	∞	0.0000	0.9942	107
	*Maricurvus*	0.87		*	0.0000	0.0030	n.d.	∞	0.0000	0.9939	111
	*Maricurvus*	0.96	*	*	0.0000	0.0013	0.827	∞	0.1429	0.9883	119
	*Maricurvus*	0.97	*		-0.0002	0.0002	0.115	∞	0.0263	0.9292	256
	*Maricurvus*	0.90	*		-0.0001	0.0005	0.402	∞	0.1649	0.9659	262
	*Maricurvus*	0.94		*	0.0000	0.0007	n.d.	∞	0.0000	0.9767	291
**B**	*Pseudomonas*	1.00	*		0.0027	0.0629	1.137	1.073	0.0140	0.0063	14
	*Pseudomonas*	0.99		*	0.0000	0.0142	n.d.	∞	0.0000	0.9961	42
	*Pseudomonas*	0.91		*	0.0000	0.0112	n.d.	∞	0.0000	0.9960	53
	Pseudomonaceae	0.82		*	0.0000	0.0045	n.d.	∞	0.0000	0.9950	88
	*Pseudomonas*	0.99		*	0.0000	0.0025	n.d.	∞	0.0000	0.9931	120
	*Pseudomonas*	0.91		*	0.0000	0.0018	n.d.	∞	0.0000	0.9913	167
	*Pseudomonas*	1.00		*	0.0000	0.0016	n.d.	∞	0.0000	0.9903	174
**C**	*Alcanivorax*	1.00	*	*	-0.0001	0.0004	0.402	∞	0.1649	0.9599	206
	*Alcanivorax*	1.00	*		-0.0007	-0.0001	0.007	-0.143	-0.0143	0.0148	270
**D**	*Methylophaga*	1.00		*	0.0000	0.0006	n.d.	∞	0.0000	0.9739	210
**E**	Rhodobacteraceae	1.00	*	*	-0.0003	0.0008	-0.167	∞	-0.1188	0.9799	104
	Rhodobacteraceae	1.00	*	*	0.0003	0.0003	1.237	∞	0.2935	0.9384	105
	Rhodobacteraceae	1.00	*	*	-0.0002	0.0001	0.115	∞	0.0263	0.7678	226
	Rhodobacteraceae	1.00		*	0.0000	0.0000	n.d.	∞	0.0000	0.6098	288

All OTUs that satisfied the Δz or ΔF criteria are listed. The first three columns show taxonomy. The most specific RDP taxonomic classification with at least 80% bootstrap support is shown. In the next two columns, asterisks (*) indicate whether the OTU satisfied the Δ*z* criteria, the Δ*F* criteria, or both. The next six columns show the changes in relative abundance (Δr.a.), rescaled reads *z*, and cumulative distribution function *F* in the control (“ct”) and experimental (“ex”) units. The value Δ*z* = n.a. is shown for OTUs that had zero counts at both timepoints in that microcosm; Δ*z* = ∞ is shown for OTUs had zero counts before the treatment and more than zero counts after the treatment.

The OTUs that appear to respond to oil are all members of γ-Proteobacteria (clades A through D) or α-Proteobacteria (clade E). Among the γ-Proteobacteria, many of these OTUs correspond to phylogenetic groups that contain known oil degraders: the two OTUs in clade C are both classified as *Alcanivorax*, the seven OTUs in clade B are classified as *Pseudomonas* or Pseudomonadaceae [36], and the one OTU in clade D is classified as *Methylophaga* [37, 38].

All but one of the eight OTUs in clade A are classified as *Maricurvus*. These seven OTUs align to NCBI entries for *Maricurvus nonylphenolicus* and *Aestuariicella hydrocarbonica*. The first species, *M*. *nonylphenolicus* is the *Maricurvus* type strain and degrades nonylphenol [39], while the second, *A*. *hydrocarbonica* has a 16S sequence highly similar to *Maricurvus* and degrades multiple aliphatic hydrocarbons [40].

The OTUs in clade E, which are members of α-Proteobacteria, are classified by RDP as Rhodobacteracea, and they align equally well to 16S sequences from *Phaeobacter*, *Roseobacter*, *Pelagimonas*, and *Sulfitobacter* spp. Although genus *Phaeobacter* has no known oil-degrading species, it may have increased in abundance in other experiments that amended ocean water with crude oil [41], and *Sulfitobacter* spp. were abundant in oiled beach sands [35] and in a large microcosm simulating an oil spill in ocean water [42].

## Discussion

### The truncated Poisson lognormal distribution and microbial ecology

As noted above, the TPL distribution has been used to model the abundance distribution of plants and animals, but to our knowledge this is the first report in which the TPL distribution is used to model microbial abundances collected in 16S surveys.

When we laid out the logic of the TPL distribution, we described the Poisson distribution as the link from the continuous-valued true abundance *λ* to the discrete number of sequencing reads. However, the number of 16S genes is not identical between organisms, and so our approach uses a single layer (a stochastic change from *λ* to the number of reads) to model a process that actually has two layers (a deterministic change from the organism’s true abundance *λ* to the true abundance of its 16S gene, and from there to reads). The quality of the TPL distribution’s fit to 16S abundance data suggests that variations in 16S copy number need not be separately included to explain the observed abundance distributions. This idea contrasts against the approach of Kembel *et al*. [17], who showed that the abundance distribution of organismal counts *N*, computed from an estimate of a taxon’s 16S copy number *C* and its 16S sequence data counts *N* × *C*, fits the lognormal better than does the abundance distribution of 16S counts *N* × *C*. Our results suggest that the lognormal is simply a poor fit for discrete 16S count data. In the ecosystems we studied, compounding the lognormal with a random Poisson distribution is sufficient to make an excellent fit to 16S OTU abundance data.

### Interpreting microbial dynamics

The information in Table 1 helps demonstrate that three quantifications of OTU dynamics—change in relative abundance, change in rescaled reads *z*, and change in cumulative distribution function value *F*—provide complementary information about the OTUs’ dynamics in our experiment. In multiple cases, OTUs satisfied both the Δ*z* and Δ*F* criteria. However, the Δ*F* criteria tend to identify organisms that are less abundant and have non-finite Δ*z* values. Some OTUs underwent a small change in relative abundance but a large change in Δ*F*, indicating the abundance distribution for 16S sequences is so skewed that that small change in relative abundance is sufficient for it to advance dramatically relative to other OTUs. For example, OTU 63 in clade A experiences a small increase in relative abundance (+0.8%) in the experimental microcosm, but its Δ*F* (+0.996 of a possible 1.0) indicates that its small change in relative abundance made it more abundant than most of the other OTUs in the sample. In this case, a small change in relative abundance for a lowly-abundant OTU can equate to a large Δ*F*. Conversely, a large change in relative abundance for a highly-abundant OTU can equate to a small Δ*F*.

We speculate that the Poisson lognormal’s fit to microbial community structure may have further relevance to making inferences about microbial community dynamics. The problem of limited replicates is not new, and difficulties in replication were probably more pronounced for microarray studies. One approach to limited replication in microarray studies was to infer the variance in each gene’s expression level, potentially improving the power of gene-by-gene *t*-tests without requiring more replicates, using Bayesian hierarchical models [43]. Just as genes with higher expression level tend to have higher variance, it may be that there are trends in the variance or dynamics of OTUs’ relative abundances. It may be that the Poisson lognormal distribution could provide a lens for discovering those trends, which in turn might provide better inference for uncovering OTUs’ dynamics if they are integrated in a productive way. It is important to again note that no method, no matter how sophisticated, can obviate the need for sufficient replication.

As mentioned in the Introduction, sequencing count data sets are compositional and are therefore subject to compositional effects. Without a quantification of overall or absolute bacterial abundance, metagenomic 16S datasets can provide evidence—but never proof—that a measured bacterial species changed in absolute abundance. However, if the Poisson lognormal distribution accurately models the distribution of OTUs’ abundances, and if most OTUs do not change in absolute abundance, then the OTUs’ positions with respect to the distribution may reflect their absolute abundances better than their relative abundances do. A well-powered experiment comparing absolute abundances and OTU’s positions in the Poisson lognormal distribution could evaluate this possible relationship.

### Possible identification of oil degraders

A simple microcosm experiment, coupled with our analytical technique, identified many OTUs that may have increased meaningfully in abundance in response to the amendment with crude oil. OTUs classified as Rhodobacteraceae had Δ*z* values indicating a small bloom, suggesting that these organisms might be involved in oil degradation without being capable of degrading oil on their own. On the other hand, the large blooms of OTUs classified as *Maricurvus*, along with the recent discovery of the closely-related oil degrader *Aestuariicella hydrocarbonica*, suggest that these organisms might be relevant to oil degradation in the Eastern Mediterranean.

### Uses and limitations for this method

In our experiment, we had enough sample material for a few microcosms, and we could non-destructively subsample each microcosm through time. Ideally, we would have had access to much more sample material so we could run many replicate experiments and use standard statistical approaches to validate any observed bacterial community dynamics. In our constrained setup, we aimed to mitigate extraneous, confounding effects like time, bottle effects, or contact with a common set of microbes by incorporating information about the community composition dynamics from multiple timepoints. Experiments in which subsampling is less onerous than replications—as in, for example, experiments studying the microbiome of animals in a facility or experiments studying the effect of a treatment *in situ*—might benefit from quantitative correction of experimental results using the data obtained from pretests and control units.

We expect that this method might also be applicable to situations beyond the very constrained one that originally motivated us. If an experimental setup has many replicates, it may be that performing rigorous statistical analyses on the Poisson lognormal metrics, rather than just the relative abundances, yields more useful results. We also expect that the Poisson lognormal distribution might fit data from amplicon-based sequencing of other taxonomic marker genes, like eukaryotes’ 18S rRNA gene.

## Supporting Information

S1 Dataset Unique sequences from sludge bioreactor experiments. Trimmed, quality filtered, dereplicated sequences.
(FASTA)

S2 Dataset Count data from sludge bioreactor experiments. Table showing number of times each sequence appeared in each sample (three replicates, two timepoints each).
(TXT)

S3 Dataset Unique sequences from oil microcosm experiments. Trimmed, merged, quality filtered, dereplicated sequences.
(FASTA)

S4 Dataset Count data from oil microcosm experiments. Table showing number of times each sequence appeared in each sample (water from four depths, control and experimental “oil” microcosms, two timepoints each).
(TXT)

S1 Fig TPL fits OTUs called by different methods. Probability-probability plots comparing the empirical cumulative distribution function (x-axis) with the theoretical cumulative probability of a TPL distribution fit to the distribution of OTUs computed using different OTU-calling methods (y-axis, black line). This is same ocean sample as in Figs 1 and 2. The first ten data points are marked with vertical dashes: the first dash (furthest lower left) represents fraction of OTUs with 1 read, the second dash represents the fraction of OTUs with 2 or fewer reads, and so forth. The dotted black line indicates a perfect fit (*y = x*). The theoretical cumulative probability of a simple lognormal distribution fit to each OTU distribution (red) is shown to emphasize the quality of the TPL fit. The methods are unique sequences (i.e., 100% identity OTUs; top left), 97% reference-based OTUs from Greengenes (top right), *de novo* 97% OTUs (bottom left), and genus-level OTUs computed with RDP (bottom right). The empirical goodness-of-fit test described in the main text yields *p* = 0.35, 0.40, 0.44, 0.41 for these data.
(EPS)

S2 Fig OTU dynamics measured by Δ*z*. Each OTU present in the microcosm experiment described in the main text is shown. OTUs that meet the Δ*z* criterion described in the text (Δ*z* in treatment > Δ*z* in treatment − 0.5) are in red. OTUs that meet the criterion and are among the 304 most abundant sequences in the four microcosm experiments (i.e., those shown as blue dots in Fig 4) are circled. OTUs with an undefined Δ*z* value in either microcosm are not shown, while OTUs with infinite Δ*z* values are shown at the border of the figure (e.g., an OTU with Δ*z* = +∞ in the control unit and -∞ in the experimental unit would be shown in the lower-left corner).
(EPS)

S3 Fig OTU dynamics measured by Δ*F*. Each OTU present in the microcosm experiment described in the main text is shown. OTUs that meet the Δ*F* criteria described in the text (Δ*F* in treatment > 0.5; Δ*F* in treatment < 0.5) are in red. OTUs that meet those criteria and are among the 304 most abundant sequences in the four microcosm experiments (i.e., those shown as red dots in Fig 4) are circled.
(EPS)
